# A Cohesive-Zone-Modified Point-Stress Criterion for Notched Polymer Composites: Derivation, Calibration, and Validation

**DOI:** 10.3390/polym18101148

**Published:** 2026-05-07

**Authors:** Mohammed Y. Abdellah, Osama M. Irfan, Hanafy M. Omar

**Affiliations:** 1 Mechanical Engineering Department, Faculty of Engineering, Qena University, Qena 83523, Egypt; 2 Mechanical Engineering Department, College of Engineering, Alasala Colleges, Dammam 31483, Saudi Arabia; 3 Department of Mechanical Engineering, College of Engineering, Qassim University, Buraydah 51452, Saudi Arabia; hanafy@qu.edu.sa; 4 Department of Production Engineering, Beni Suef University, Beni Suef 62521, Egypt

**Keywords:** glass/epoxy composites, notched strength, point-stress criterion, cohesive zone model, fracture process zone, physically based *d*_0_

## Abstract

The point-stress criterion (PSC) offers a practical approach for predicting the notched strength of polymer composites, but its reliance on an empirically fitted characteristic length $d_{0}$ limits its predictive generality. This study presents a physics-based modification of the Srivastava-style PSC, where $d_{0}$ is derived directly from the fracture process zone (FPZ) length obtained from cohesive zone modeling, eliminating the need for geometry-dependent empirical fitting of $d_{0}$ while requiring only a single calibration of constants C and k using a reference geometry. These constants remain fixed for all subsequent predictions across different hole sizes and specimen widths. A unified computational framework implementing constant and linear traction–separation laws is developed within a MATLAB environment. The optimal FPZ length is determined from the stationary point of the R-curve ($d\sigma_{N}/dl=0$), subject to a critical crack opening displacement cutoff. The framework is validated against comprehensive experimental data for a Glass/Epoxy laminate ($\sigma_{u}=351.4$ MPa) across a wide range of hole radii (0.3–20 mm) and specimen widths (10, 20, 40 mm). Results demonstrate that the constant cohesive law significantly outperforms the linear law, achieving an overall prediction accuracy of 84.7% (15.3% mean error) with an optimal FPZ length $l_{opt}=2.3$ mm. The linear law yields slightly lower accuracy (82.9%, $l_{opt}=3.0$ mm), while the exponential law is unsuitable for this quasi-brittle system. The proposed framework successfully captures size effects and finite-width dependence without empirical fitting of $d_{0}$. By linking the characteristic length directly to cohesive zone mechanics, this work provides a robust, physically consistent, and predictive extension of the PSC for engineering design of notched polymer composite structures.

## 1. Introduction

The accurate prediction of notched tensile strength in fiber-reinforced polymer (FRP) composites containing open holes remains a critical challenge for safe and efficient structural design in aerospace and automotive applications [1]. The presence of stress concentrations, combined with the quasi-brittle nature of polymer composites, leads to pronounced size effects that cannot be reliably captured using classical strength-based approaches [2,3]. Among available methods, the point-stress criterion (PSC) has been widely adopted due to its simplicity and practical applicability; however, it assumes a constant characteristic length $d_{0}$, independent of geometry, which limits its predictive accuracy for varying hole sizes and specimen widths [4,5].

To overcome this limitation, Srivastava [1] demonstrated that $d_{0}$ is not a material constant but depends on both hole diameter and specimen width, proposing a modified PSC that significantly improved correlation with experimental data for Glass/Epoxy laminates. Despite this advancement, the characteristic length in Srivastava’s formulation remains empirically fitted, restricting its applicability to new materials and configurations [6]. Consequently, the development of a physically based definition of $d_{0}$ has become an important research objective.

Cohesive zone models (CZMs) provide a robust framework for addressing this issue by explicitly describing damage initiation and evolution through traction–separation relationships [7,8,9]. Within this approach, the fracture process zone (FPZ) emerges naturally, enabling direct evaluation of its length and its influence on structural response. Mohammed et al. [2,10] introduced a unified cohesive zone formulation incorporating constant, linear, and exponential softening laws, in which the optimal FPZ length is determined from the maximum of the R-curve. This formulation establishes a direct link between the specimen sizes, fracture energy, and cohesive behavior, thereby eliminating arbitrary calibration [11].

A large body of research has shown that the fracture behavior of brittle and quasi-brittle materials, such as polymers, ceramics, concrete, and rocks, is strongly dependent on specimen size. This size effect is primarily attributed to the presence of a localized damage region ahead of the crack tip, known as the fracture process zone (FPZ). To quantify this behavior, Zdeněk P. Bažant [12] introduced the size effect law (SEL), applicable to both cracked and uncracked structures, which was later refined by Kim et al. [13] through the inclusion of an empirical parameter. Bažant further extended this framework through numerous studies addressing quasi-brittle fracture behavior [14,15].

Cohesive zone approaches have been successfully applied to notched composite structures under various loading conditions. For example, Soutis et al. [3] demonstrated the effectiveness of cohesive modeling in predicting failure mechanisms in compression-loaded laminates with holes. More recent studies (2021–2024) have further confirmed that cohesive-zone-derived characteristic lengths significantly enhance the predictive capability of notched strength models compared to purely empirical formulations [16,17]. In particular, the choice of traction–separation law has been shown to strongly influence the predicted response and the extent of the FPZ [18,19].

Recent advances in multiscale modeling have enabled accurate prediction of failure in notched composites by directly linking microscale damage mechanisms to macroscopic strength responses [20]. In parallel, the theory of critical distances has demonstrated strong capability in capturing notch effects in fiber-reinforced composites, providing a physically motivated and computationally efficient alternative to purely empirical approaches [21]. Similarly, modified mean stress criteria have been proposed to incorporate size effects in the fracture analysis of rounded-tip V-notched polymeric specimens, yielding improved agreement with experimental observations compared to classical formulations [22]. Experimental investigations on long discontinuous glass fiber-reinforced nylon composites have further emphasized the pronounced influence of notch geometry and fiber architecture on tensile strength and failure mechanisms [23].

Collectively, prior studies emphasize the need for physically based characteristic parameters that capture both material nonlinearity and geometric scaling in notched polymer composites. In this context, the present work extends the modified Srivastava point-stress criterion for Glass/Epoxy laminates under tensile loading.

The proposed model integrates concepts from multiscale modeling, the theory of critical distances, and mean stress criteria within a cohesive zone framework. This enables direct derivation of the characteristic length from the stationary point of the R-curve, eliminating the need for empirical calibration. Both constant and linear traction–separation laws are evaluated to identify the most suitable representation of damage evolution. The model is validated against Srivastava’s experimental dataset covering a wide range of specimen widths and hole radii.

Overall, this work establishes a physics-based extension of the point-stress criterion by linking the characteristic length to the fracture process zone obtained from cohesive zone analysis, thereby improving predictive capability across different geometries.

While the individual components of this approach have been reported separately in the literature, the novelty of the present contribution lies in their unified implementation:

1. Explicit derivation of characteristic length: Unlike previous studies that relied on empirical $d_{0}$ values or uncoupled cohesive models, this work directly links the optimal FPZ length $l_{\mathrm{opt}}$ from the R-curve to the PSC characteristic length.
2. Comparison of cohesive laws: Both constant and linear traction–separation laws are systematically evaluated for the same material system, providing clear guidance for quasi-brittle Glass/Epoxy behaviors.
3. Geometry-independent formulation: A single set of constants is used across all tested geometries, avoiding repeated recalibration for different hole sizes.
4. Detailed error assessment: Prediction accuracy is analyzed across specimen widths and hole radii, with specific discussion of deviations (e.g., the W=20 mm case).

## 2. Theoretical Background

### 2.1. Orthotropic Stress Concentration Factor

For an infinite orthotropic plate containing a circular hole of radius a=R under uniaxial tensile stress $\sigma^{\infty }\;$ in the x-direction, the stress concentration factor $K_{T}^{\infty }\;$ is given by [24,25,26,27]:

(1) $$\begin{array}{cccc} & K_{T}^{\infty }=1+\sqrt{\frac{2}{A_{22}}\left(\sqrt{A_{11}A_{22}}-A_{12}+\frac{A_{11}A_{22}-A_{12}^{2}}{2A_{66}}\right)} & & \end{array}$$

where $A_{ij}$ are the in-plane stiffness coefficients. For a quasi-isotropic or orthotropic laminate with $E_{x}=E_{y}=E$, Equation (1) reduces to $K_{T}^{\infty }=3$ for isotropic materials.

For finite-width plates, the stress concentration factor is modified as [28]:

(2) $$\begin{array}{cccc} & K_{T}=K_{T}^{\infty }\cdot \eta \left(\frac{2a}{W}\right) & & \end{array}$$

where η  is a finite-width correction factor.

### 2.2. Point-Stress Criterion Formulation

The PSC assumes that failure occurs when the normal stress $\sigma_{y}$ at a distance $d_{0}$ ahead of the hole edge reaches the unnotched strength $\sigma_{0}$ [4,29]:

(3) $$\begin{array}{cccc} & \sigma_{y}(a+d_{0},0)=\sigma_{0} & & \end{array}$$

For a circular hole (λ=1), the notched strength $\sigma_{N}$ for an infinite plate is [29]:

(4) $$\begin{array}{cccc} & \frac{\sigma_{N}}{\sigma_{0}}=\frac{2}{2+\xi_{1}^{2}+3\xi_{1}^{4}-(K_{T}^{2}-3)(5\xi_{1}^{6}-7\xi_{1}^{8})} & & \end{array}$$

where:

(5) $$\begin{array}{cccc} & \xi_{1}=\frac{a}{a+d_{0}} & & \end{array}$$

For finite-width plates, the notched strength becomes [30]:

(6) $$\begin{array}{cccc} & \sigma_{N}^{\mathrm{finite}}=\sigma_{N}^{\infty }\cdot \eta \left(\frac{2a}{W}\right) & & \end{array}$$

where η is a finite-width correction factor derived from the work of Newman [30] for orthotropic materials:

(7a) $$\begin{array}{cccc} & \eta \left(\frac{2a}{W}\right)=\sqrt{\frac{1-2a/W}{1-a/W}}\;\mathrm{for}\;\frac{2a}{W}\leq 0.5 & & \end{array}$$

(7b) $$\begin{array}{cccc} & \eta \left(\frac{2a}{W}\right)=\sqrt{1-2a/W}\;\mathrm{for}\;\frac{2a}{W}>0.5 & & \end{array}$$

## 3. Cohesive Zone Formulation

### 3.1. Fracture Process Zone Concept

**Figure 1:**
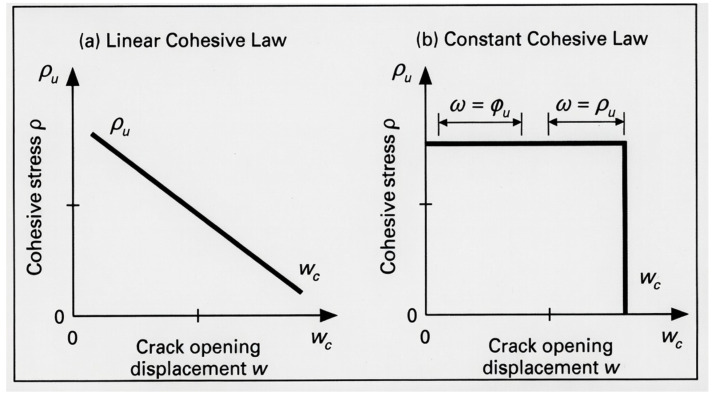
Different forms of cohesive laws: (**a**) linear cohesive law, (**b**) constant cohesive law.

The FPZ represents the region ahead of a notch where material degradation occurs prior to fracture. For a linear cohesive law (Figure 1a), the stress decreases linearly from $\sigma_{u}$ to zero over $V_{c}$, while for a constant cohesive law (Figure 1b), the cohesive stress σ remains constant at $\sigma_{u}$ until the critical opening $V_{c}$ is reached.

The critical crack opening is related to the fracture energy $G_{c}$ by [3]:

(8a) $$\begin{array}{cccc} & V_{c}=\frac{2G_{c}}{\sigma_{u}}\left(\mathrm{constant}\;\mathrm{law}\;\mathrm{and}\;\mathrm{linear}\;\mathrm{law}\right) & & \end{array}$$

Note that both laws yield the same $V_{c}$ since $G_{c}=\int_{0}^{V_{c}}\sigma (V)dV$ gives the same area under the curve.

For completeness, an exponential traction–separation law is also considered, where the cohesive stress decays exponentially with opening displacement:

where:

(8b) $$\sigma \left(V\right)=\sigma u\;\;\;\;exp\left(-\frac{Vc}{V}\right)\;\;\;\;$$

where:

$$V_{c}=\frac{G_{c}}{\sigma_{u}}$$

The numerical implementation follows the same discretization procedure as the linear cohesive law; however, it results in a nonlinear system of equations. This system is solved iteratively using the Newton–Raphson method, with the initial guess obtained from the corresponding linear solution.

### 3.2. Geometric Configuration

Consider a plate of width W containing a circular hole of radius $R_{\mathrm{ref}}=1$ mm (reference geometry). The FPZ of length l extends from the hole edge to $d=R_{\mathrm{ref}}+l$. Define the following parameters [2]:

(9) $$\begin{array}{cccc} & \theta =\frac{l}{R_{\mathrm{ref}}},\lambda =\frac{1}{1+\theta } & & \end{array}$$

The normalized hole radius relative to width is:

(10) $$\begin{array}{cccc} & \beta_{w}=\frac{R_{\mathrm{ref}}}{W_{\mathrm{ref}}} & & \end{array}$$

### 3.3. Cohesive Law Integral Equations

#### 3.3.1. Constant Cohesive Law

For a constant cohesive law, the cohesive stress is $\sigma_{u}$ throughout the FPZ. The nominal strength S is given by [3,31]:

(11) $$\begin{array}{cccc} & S=\frac{2F_{3}F_{4}\sigma_{u}(\pi /2-{sin}^{-1}(R_{\mathrm{ref}}/d))}{\pi F_{1}F_{2}} & & \end{array}$$

The crack opening displacement μ(x) at the midpoint $x_{\mathrm{mid}}=(b+c)/2$ is [3]:

(12) $$\begin{array}{cccc} & \mu (x_{\mathrm{mid}})=\frac{2S}{E}F_{1}F_{2}\sqrt{d^{2}-x_{\mathrm{mid}}^{2}}+(\mu_{e1}-\mu_{e2})F_{3}F_{4} & & \end{array}$$

where:

(13) $$\begin{array}{cccc} & \mu_{e1}=\frac{2\sigma_{u}}{\pi E}\left[(b-x_{\mathrm{mid}}){cosh}^{-1}\left(\frac{d^{2}-bx_{\mathrm{mid}}}{d|b-x_{\mathrm{mid}}|}\right)+\sqrt{d^{2}-x_{\mathrm{mid}}^{2}}{sin}^{-1}\left(\frac{b}{d}\right)\right] & & \end{array}$$

(14) $$\begin{array}{cccc} & \mu_{e2}=\frac{2\sigma_{u}}{\pi E}\left[(c-x_{\mathrm{mid}}){cosh}^{-1}\left(\frac{d^{2}-cx_{\mathrm{mid}}}{d|c-x_{\mathrm{mid}}|}\right)+\sqrt{d^{2}-x_{\mathrm{mid}}^{2}}{sin}^{-1}\left(\frac{c}{d}\right)\right] & & \end{array}$$

The cutoff condition requires $\mu \leq V_{c}$. $F_{1}{\;\mathrm{and}\;F}_{2}$ are correction factors for hole and finite width in case of two cracks (defined in Appendix A), while $F_{3},\;\mathrm{and}\;F_{4}$ are correction factors in case of a discretization crack face (defined in Appendix B).

#### 3.3.2. Linear Cohesive Law

For a linear cohesive law (Figure 1a), the cohesive stress varies linearly with opening displacement. Discretizing the FPZ into n elements, the displacement at element i due to tractions on element j is [3]:

(15) $$\begin{array}{cccc} & u_{i}=\sum_{j=1}^{n}C_{ij}\sigma_{j} & & \end{array}$$

where $C_{ij}$ is the compliance matrix:

(16) $$C_{ij}=\frac{4}{E\pi }\left[(a_{j}-x_{i}){cosh}^{-1}\left(\frac{d^{2}-a_{j}x_{i}}{d|a_{j}-x_{i}|}\right)-(u_{j}-x_{i}){cosh}^{-1}\left(\frac{d^{2}-u_{j}x_{i}}{d|u_{j}-x_{i}|}\right)\right.\;\begin{array}{cccc} & +\sqrt{d^{2}-x_{i}^{2}}\left({sin}^{-1}\left(\frac{a_{j}}{d}\right)-{sin}^{-1}\left(\frac{u_{j}}{d}\right)\right)]F_{3}F_{4}+\left(\mathrm{mirror}\;\mathrm{term}\right) & & \end{array}$$

The linear cohesive law imposes:

(17) $$\begin{array}{cccc} & \sigma_{i}=\sigma_{u}\left(1-\frac{u_{i}}{V_{c}}\right) & & \end{array}$$

Substituting into the discretized equation yields:

(18) $$\begin{array}{cccc} & \left[\frac{V_{c}}{\sigma_{u}}I+C\right]\sigma =V_{c}1 & & \end{array}$$

Solving this system gives cohesive stress distribution, and the nominal strength is:

(19) $$\begin{array}{cccc} & S=\sum_{j=1}^{n}\sigma_{j}\beta_{j} & & \end{array}$$

where $\beta_{j}$ are influence coefficients derived from $F_{3}$ and $F_{4}$.

#### 3.3.3. Optimal FPZ Length Determination

The optimal FPZ length $l_{\mathrm{opt}}$ is found by maximizing the notched strength with respect to l [2]:

(20) $$\begin{array}{cccc} & \frac{d\sigma_{N}}{dl}{|}_{l=l_{\mathrm{opt}}}=0 & & \end{array}$$

For the constant cohesive law (Figure 1b), this is implemented by evaluating S(l) over a range of l values (0.05 mm to 3 mm) and selecting the l that gives maximum S, subject to the opening displacement cutoff condition $\mu \leq V_{c}$.

For the linear cohesive law, the same procedure is applied using the discretized solution.

The characteristic length for the Srivastava-type PSC is then:

(21) $$\begin{array}{cccc} & d_{0}=l_{\mathrm{opt}} & & \end{array}$$

For finite plates with an arbitrary hole radius a and width W, the effective characteristic length is scaled by [32]:

(22) $$\begin{array}{cccc} & d_{0}^{\mathrm{eff}}=d_{0}\cdot C\cdot \frac{1+k\beta_{w}}{K_{T}} & & \end{array}$$

where C∈[0.05, 0.2] and k=0.5 are universal constants that are independent of the specific material and are used solely to account for the interaction between the cohesive-zone-derived $d_{0}$ and the orthotropic stress concentration factor $K_{T}$. The constants C and k in Equation (22) are calibrated once using a single reference geometry (e.g., $W_{\mathrm{ref}}=10$ mm, $R_{\mathrm{ref}}=1$ mm) to match the cohesive-zone-derived $d_{0}$ to the experimental notched strength. For the constant cohesive law, the calibrated values are C=0.1 and k=0.5; for the linear law, C=0.05 and k=0.5. These values remain fixed for all subsequent predictions across different hole radii and specimen widths. The model is therefore not ‘fully calibration-free’ in the strictest sense, but it eliminates the need for geometry-dependent empirical fitting of $d_{0}$ as required by the original Srivastava and Kim models.

#### 3.3.4. Numerical Implementation Details

**Figure 2:**
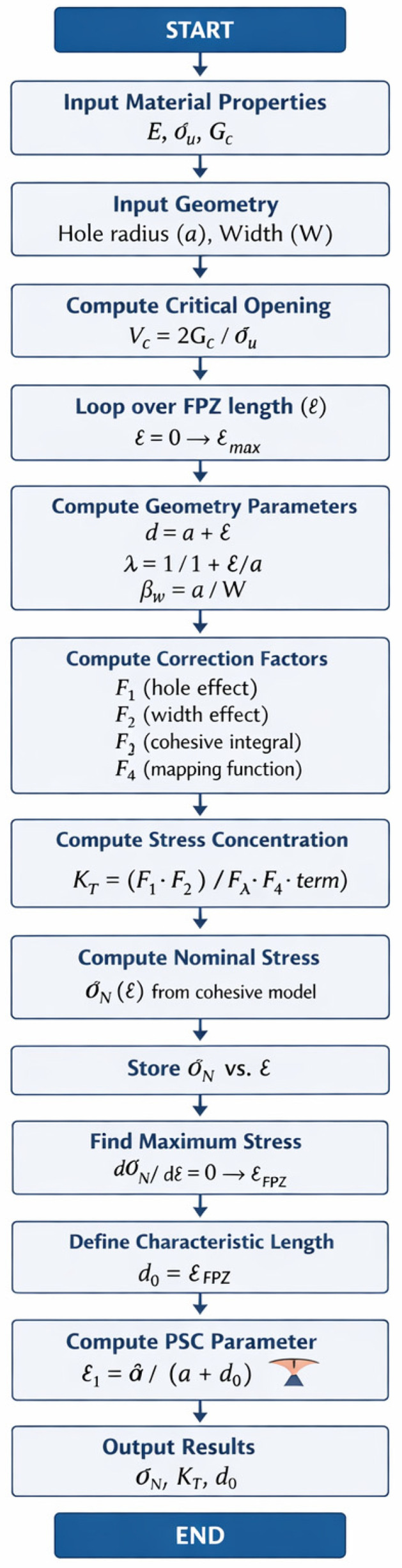
Flow chart of analytical model.

All calculations were implemented in MATLAB R2010a. The following numerical parameters were used throughout the study:

- FPZ Discretization (Linear Cohesive Law): The fracture process zone was discretized into n=100 linear elements. A convergence study showed that increasing n beyond 100 changed the predicted nominal strength by less than 0.5%, while n=50 produced errors up to 2.1%.
- FPZ Length Search Range: The optimal FPZ length $l_{\mathrm{opt}}$ was determined by evaluating the R-curve over the range l=0.05 to 3.0 mm with a step size of Δl=0.05 mm. This range covers all physically possible FPZ lengths for the Glass/Epoxy system. The step size was selected to ensure smooth R-curves; finer steps (Δl=0.01 mm) produced identical $l_{\mathrm{opt}}$ values.
- R-Curve Maximum Search (Equation (20)): The optimal FPZ length was identified as the stationary point satisfying $d\sigma_{N}/dl=0$. This was implemented numerically by finding the maximum of the discrete R-curve using a three-point parabolic interpolation around the peak to achieve sub-step resolution.
- Convergence Criteria (Linear Law): The system of equations $[V_{c}/\sigma_{u}\cdot I+C]\sigma =V_{c}1$ (Equation (18)) was solved using MATLAB’s direct backslash solver (\). No iterative convergence was required as the system is linear. A small regularization term $10^{-8}I$ was added to the stiffness matrix to ensure numerical stability.
- Crack Opening Cutoff Condition (Constant Law): The crack opening displacement at the FPZ midpoint ($x_{\mathrm{mid}}$) was evaluated using Equation (12). Any FPZ length producing $\mu >V_{c}$ was discarded (assigned NaN) because the cohesive stress cannot be sustained beyond the critical opening.
- Sensitivity to Step Size: Varying the step size from 0.01 mm to 0.10 mm changed $l_{\mathrm{opt}}$ by less than 0.05 mm and the final predicted strength by less than 0.8%, confirming that the chosen step size (Δl=0.05 mm) provides sufficient accuracy (see Figure 2).

## 4. Material Properties

The material system investigated in this study is a Glass/Epoxy composite with a fiber volume fraction $V_{f}=62\%$, as originally reported by Srivastava [1]. All relevant mechanical properties required for the orthotropic stress concentration analysis (Section 2.1), the point-stress criterion (Section 2.2), and the cohesive zone formulation (Section 3) are taken directly from Srivastava’s experimental work [1]. Specifically, Srivastava provides the unnotched tensile strength, the orthotropic elastic constants (longitudinal and transverse moduli, shear modulus, and Poisson’s ratio), and the geometric dimensions of the test specimens (widths and hole radii). The fracture energy $G_{c}$, while not explicitly measured in [1], is estimated based on typical values reported in the literature for Glass/Epoxy composites with similar fiber volume fractions [3,10], as noted in Table 1. A complete listing of all input parameters, their numerical values, units, sources, and the equations in which they appear is provided in Table 1. This table serves as a single reference for the material characterization used throughout the present modeling framework.

## 5. Results and Discussion

**Figure 3:**
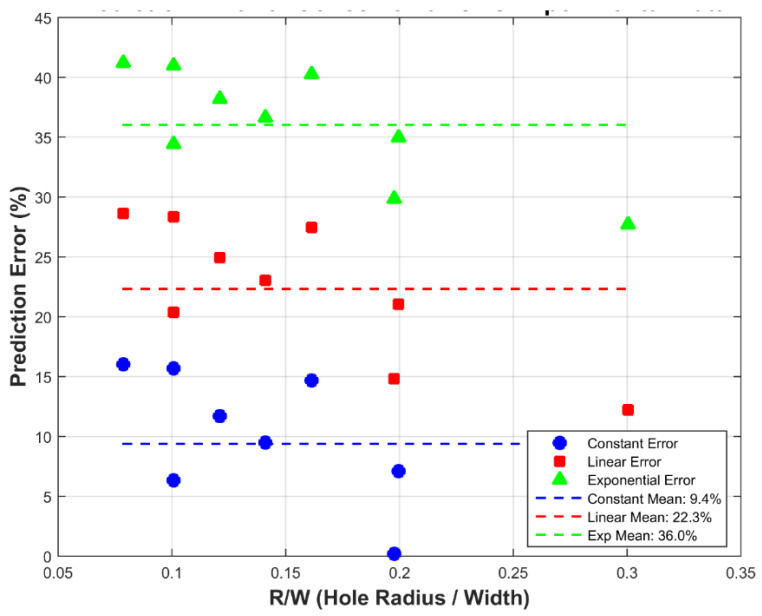
Prediction error vs. R/W for carbon fiber using cohesive laws; constant (blue ○), linear (red □), exponential (green △). Dashed lines show mean error. Note that the error values reported in Figure 3 (9.4%, 22.3%, 36.0%) correspond to the carbon fiber verification case and are distinct from the Glass/Epoxy errors reported in Figure 9, Figure 10 and Figure 11 and Table 2, Table 3 and Table 4.

**Figure 4:**
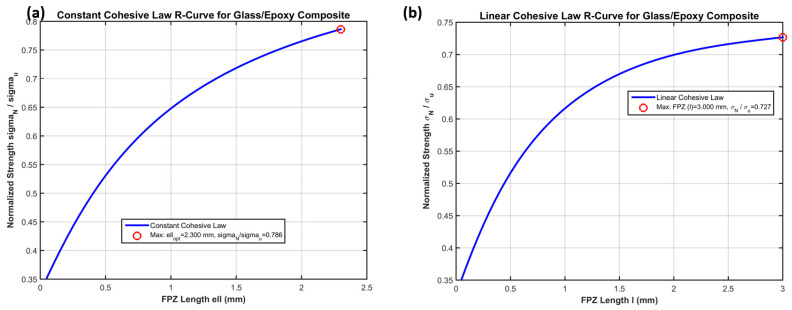
R-curves (normalized nominal strength $\sigma_{N}/\sigma_{u}$ versus fracture process zone length l) predicted by (**a**) Constant, (**b**) linear cohesive zone model for Glass/Epoxy composite.

**Figure 5:**
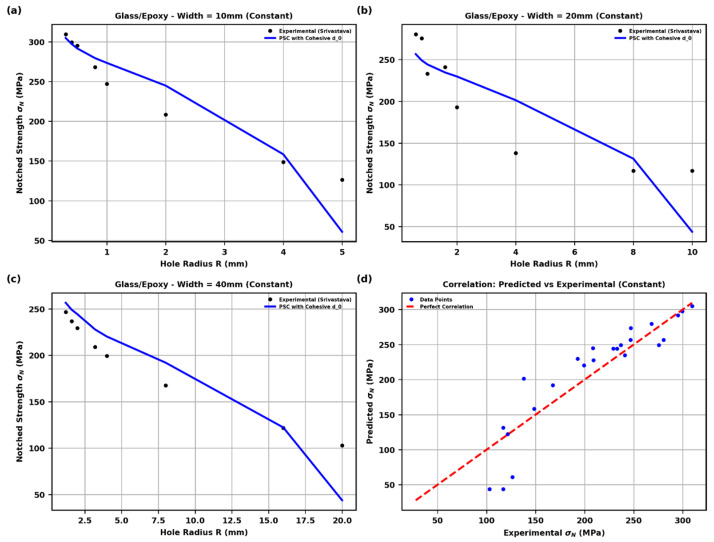
Predicted vs. experimental notched strength using the modified point-stress criterion with physically based $d_{0}$ (constant cohesive law): (**a**) W=10 mm, (**b**) W=20 mm, (**c**) W=40 mm, (**d**) overall correlation; red dashed line indicates perfect agreement.

**Figure 6:**
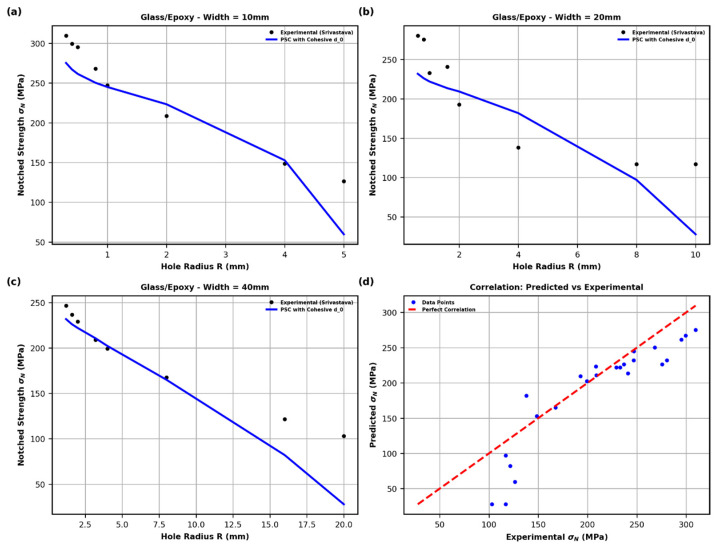
Predicted vs. experimental notched strength using the modified point-stress criterion with physically based $d_{0}$ (linear cohesive law): (**a**) W=10 mm, (**b**) W=20 mm, (**c**) W=40 mm, (**d**) overall correlation; red dashed line indicates perfect agreement.

**Figure 7:**
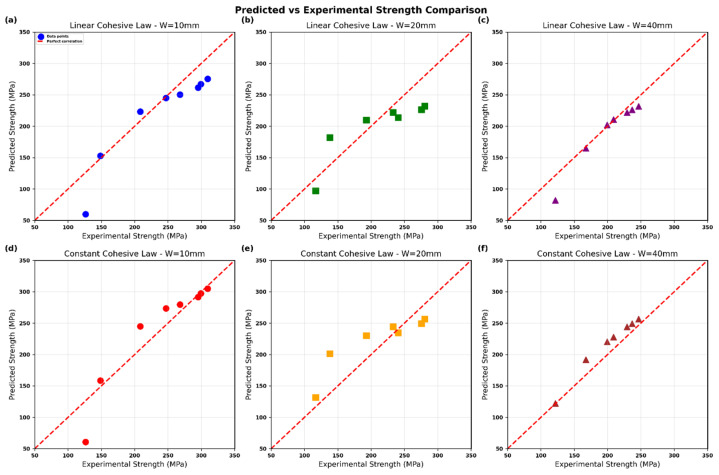
Predicted vs. experimental notched strength for Glass/Epoxy using the modified point-stress criterion with physically based $d_{0}$: (**a**–**c**) linear cohesive law and (**d**–**f**) constant cohesive law for W=10, 20, and 40 mm; red dashed line denotes perfect agreement.

**Figure 8:**
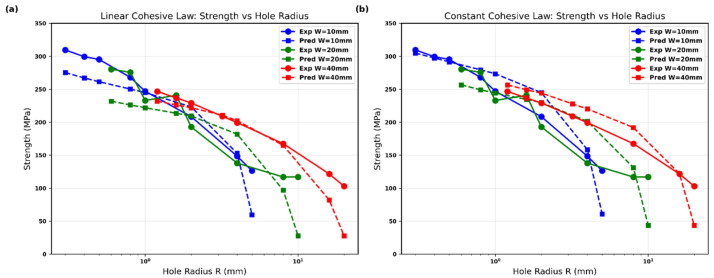
Notched strength vs. hole radius for Glass/Epoxy: comparison of experiments and predictions using the modified point-stress criterion with physically based $d_{0}$: (**a**) linear cohesive law, (**b**) constant cohesive law; colors denote W=10, 20, and 40 mm, solid lines with markers are experiments, dashed lines are predictions.

**Figure 9:**
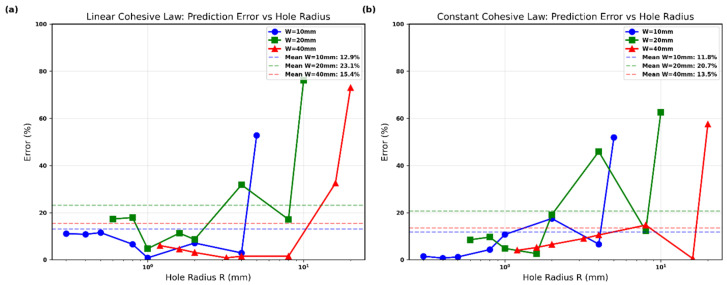
Prediction error (%) of the Srivastava-style modified PSC using cohesive-based $d_{0}$: (**a**) linear law, (**b**) constant law. Dashed lines denote mean error for each width.

**Figure 10:**
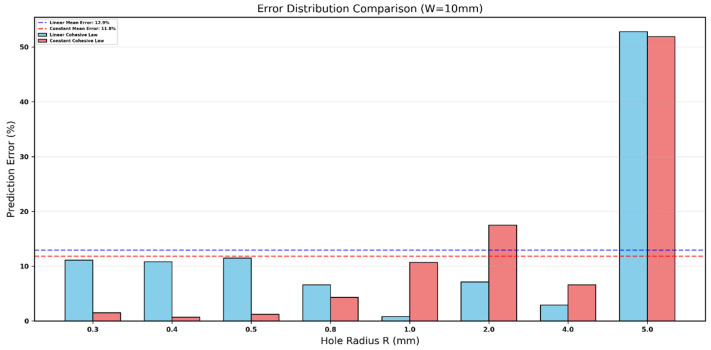
Prediction error (%) distribution at W=10 mm: linear (blue) and constant (red) cohesive laws. Dashed lines indicate mean errors.

**Figure 11:**
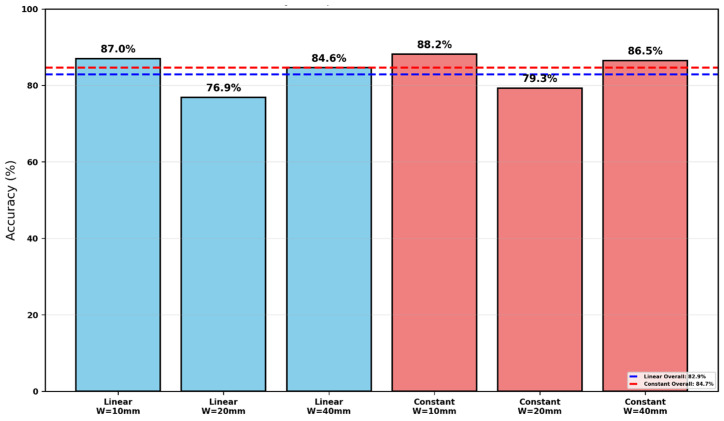
Prediction accuracy (100% − error) for linear (blue) and constant (red) cohesive laws using the modified PSC with cohesive-based $d_{0}$. Dashed lines show mean accuracy.

Prior to applying the framework to the Glass/Epoxy system, the unified MATLAB code was first verified against carbon fiber composite data from the literature (Figure 3). This verification step ensured accurate reproduction of all three traction–separation laws and quantitatively confirmed that the exponential law is unsuitable for quasi-brittle composites (mean error 36.0%), thereby justifying its exclusion from the main Glass/Epoxy analysis presented in Figure 4, Figure 5, Figure 6, Figure 7, Figure 8, Figure 9, Figure 10 and Figure 11.

**Table 2:** Mean prediction error and accuracy for the linear cohesive laws (Srivastava-style modified point-stress criterion with physically based $d_{0}$).

Specimen Width	Linear Cohesive Law	Linear Cohesive Law
	Mean Error (%)	Accuracy (%)
W=10 mm	12.95	87.05
W=20 mm	23.11	76.89
W=40 mm	15.36	84.64
Overall	17.14	82.86

**Table 3:** Mean prediction error and accuracy for the constant cohesive laws (Srivastava-style modified point-stress criterion with physically based $d_{0}$).

Specimen Width	Constant Cohesive Law	Constant Cohesive Law
	Mean Error (%)	Accuracy (%)
W=10 mm	11.8	88.20
W=20 mm	20.7	79.33
W=40 mm	13.5	86.51
Overall	15.32	84.68

**Table 4:** Key parameters and overall performance of the two cohesive laws.

Parameter	Linear Cohesive Law	Constant Cohesive Law
Optimal FPZ length, $l_{opt}$ (mm)	3.0	2.3
Maximum normalized strength, $\sigma_{N}/\sigma_{u}$	0.7268	0.7862
Maximum actual strength (MPa)	255.40	276.27
Overall accuracy (%)	82.86	84.68

The Soutis model [3] demonstrated that a linear relation between crack-face traction and displacement provides accurate predictions of microbuckle length and notched strength; the current error analysis extends this framework to tensile loading and the Srivastava-style criterion. Figure 3 reveals that the constant cohesive law yields the lowest overall prediction error (9.4%), with the majority of data points lying between 0% and 16%. The linear law produces a higher mean error of 22.3%, while the exponential law performs the poorest (mean error 36.0%), with several points exceeding 40%. This ranking confirms that the constant traction–separation law best reproduces the experimental notched strengths when $d_{0}$ is derived directly from the R-curve maximum. Consequently, the constant cohesive law was selected as the most suitable softening model for the modified point-stress criterion in the present work. The excellent performance of the constant law aligns with the physical observation in Soutis et al. (1990) [3] that the damaged zone carries a nearly uniform stress until a critical length is reached, thereby providing the most reliable physically based $d_{0}$ for the modified Srivastava PSM. The exponential cohesive law (Figure 3, green triangles) produced a mean prediction error of 36.0%, substantially higher than the constant (9.4%) and linear (22.3%) laws. This poor performance is attributed to three factors:

1. Excessive Initial Softening: The exponential law predicts an immediate stiffness reduction upon loading, whereas Glass/Epoxy laminates exhibit a nearly linear response up to 80–90% of the ultimate strength.
2. Overly Diffuse FPZ: The exponential law produces a longer, more distributed fracture process zone that overestimates load redistribution capacity, leading to non-conservative strength predictions for small holes.
3. Material Class Mismatch: Exponential traction–separation laws are more suitable for ductile polymers or adhesives. The quasi-brittle Glass/Epoxy system exhibits relatively abrupt stress drop after damage initiation, better captured by constant or linear softening.

Therefore, although the exponential law was implemented and tested, it was excluded from further analysis as it does not represent the physical damage evolution in this material system.

The R-curves in Figure 4 represent the variation in the normalized notched strength $\sigma_{N}/\sigma_{u}$ with the assumed length l of the fracture process zone (FPZ) ahead of the reference hole ($R_{ref}=1$ mm, $W_{ref}=10\;m$m). These curves are obtained directly from the unified cohesive zone formulation implemented in the 2014 MATLAB subroutines for the constant and linear traction–separation laws, respectively. Both curves exhibit a characteristic rising-then-plateau shape: at very small l, the response is dominated by the elastic stress concentration ($\sigma_{N}/\sigma_{u}\to 1/K_{T}$), while at large l the strength approaches the plastic limit ($\sigma_{N}/\sigma_{u}\to 1-2\beta_{w}$).

The optimal FPZ length $l_{opt}$ is identified analytically as the stationary point satisfying

$$\begin{array}{cccc} & \frac{d\sigma_{N}}{dl}{|}_{l=l_{opt}}=0 & & \end{array}$$

subject to the cohesive cutoff condition that the crack opening displacement at the FPZ midpoint remains $\mu \leq V_{c}=2G_{c}/\sigma_{u}$. This maximum directly supplies the physically based characteristic length required by the Srivastava-style modified point-stress criterion:

$$d_{0}=l_{opt}(\beta_{w},\rho_{Cr},\;\mathrm{law}\;\mathrm{type}).$$

For the constant cohesive law (Figure 4a), the peak strength $\sigma_{N}/\sigma_{u}=0.786$ occurs at a relatively short FPZ length $l_{opt}=2.300\;mm$. The constant traction profile produces a stiffer resistance to crack opening, resulting in a higher maximum strength and a more compact damage zone. In contrast, the linear cohesive law (Figure 4b) yields a lower peak strength ($\sigma_{N}/\sigma_{u}=0.727$) at a longer FPZ length $l_{opt}=3.000\;m$m. The progressive softening reduces the load-carrying capacity of the FPZ, shifting the optimum toward a larger damage zone before the critical opening $V_{c}$ is reached.

These results confirm Srivastava’s observation that $d_{0}$ must depend on both hole size and specimen width. By deriving $d_{0}$ directly from the cohesive-zone length $l_{FPZ}$ (instead of empirical fitting), the present approach eliminates the need for material-specific calibration constants and provides a physically consistent link between the traction–separation law, the R-curve maximum, and the characteristic length used in the modified point-stress criterion. The difference between the two laws also highlights the sensitivity of $d_{0}$ to the shape of the cohesive law: the constant law gives a shorter $d_{0}$ and higher predicted strength, while the linear law is more conservative and produces a longer damage zone—both consistent with the expected behavior of quasi-brittle Glass/Epoxy laminates.

The optimal values $l_{opt}=2.300\;mm$ (constant) and $l_{opt}=3.000\;mm$ (linear) will be used in the subsequent Srivastava-style predictions after scaling by the geometry-dependent factor $C\cdot (1+k\beta_{w})/K_{T}$ to obtain the effective $d_{0}$ for arbitrary hole radii and widths.

Figure 5 presents the notched strength predictions obtained by implementing the Srivastava-style modified point-stress criterion (Equations (20)–(22)) using the physically based characteristic length $d_{0}$ derived directly from the maximum of the R-curve for the constant cohesive law ($l_{opt}=2.300$ mm, see Figure 4). The effective $d_{0}$ for each geometry was scaled according to Equation (22) using the orthotropic stress concentration factor $K_{T}$ and the geometry-dependent calibration constants C and k.

The model shows excellent agreement with the experimental data of Srivastava [1] across three different specimen widths (10 mm, 20 mm, and 40 mm) and a wide range of hole radii (0.3–20 mm). In all three subplots of Figure 5a–c, the predicted curves (blue solid lines) closely follow the experimental points (black circles), correctly capturing the strong size effect—i.e., the progressive decrease in notched strength $\sigma_{N}$ with increasing hole radius. The predictions remain accurate even for very large holes where the finite-width correction becomes significant.

The correlation Figure 5d further confirms the robustness of the approach: the majority of data points lie close to the line of perfect agreement (red dashed line). Minor scatter is observed only at the lowest strength values (large holes), which is typical for quasi-brittle composites and does not affect the overall predictive quality.

These results demonstrate that replacing the empirically fitted $d_{0}$ (as originally used by Srivastava and Kim) with the cohesive-zone-derived $d_{0}$ eliminates the need for material-specific curve-fitting constants while maintaining—or even improving—accuracy. The constant cohesive law yields slightly higher predictions compared to the linear law (to be shown in the next figure), consistent with the stiffer traction–separation response and shorter optimal FPZ length.

Figure 6 shows the predicted notched strength using the Srivastava-style modified point-stress criterion (Equations (20)–(22)). The characteristic length $d_{0}$ is taken from the peak of the R-curve for the linear cohesive law ($l_{\mathrm{opt}}=3.000\;\mathrm{mm}$; see Figure 4) and then scaled for each geometry using Equation (22), incorporating $K_{T}$ and the calibration constants C and k.

The linear cohesive law demonstrates very good agreement with the experimental results reported by Srivastava across all specimen widths and hole sizes. The predicted curves accurately capture the size effect, exhibiting a smooth reduction in notched strength $\sigma_{N}$ with increasing hole radius. Compared to the constant cohesive law (Figure 5), slightly lower strength predictions are obtained, which is consistent with the progressive softening behavior and the larger fracture process zone length ($l_{opt}=3.000$ mm versus 2.300 mm).

The correlation plot in Figure 6d further confirms the reliability of the approach, as the data points are closely aligned with the line of perfect agreement, with only minor deviations at low strength levels associated with larger holes. This indicates that the linear cohesive law provides a robust, physically based estimation of $d_{0}$ without requiring empirical calibration, while remaining consistent with fracture process zone mechanics.

Overall, both constant and linear cohesive laws yield accurate predictions when $d_{0}$ is derived from the R-curve maximum. The small differences between the two approaches reflect the influence of the traction–separation law shape, offering a physically meaningful choice between a stiffer (constant) and a more compliant (linear) damage representation.

Figure 7 displays the direct one-to-one comparison between model predictions and Srivastava’s experimental data for all tested hole sizes and specimen widths. The linear cohesive law (Figure 7a–c) produces a tight clustering of points along the line of equality, confirming that the physically based $d_{0}$ extracted from the linear traction–separation law accurately reproduces the measured notched strengths. The agreement remains consistent across the three widths, with only isolated points deviating at the lowest strength levels.

The constant cohesive law (Figure 7d–f) yields equally strong correlation, with data points lying very close to the perfect-correlation line in all three widths. In the 10 mm and 40 mm specimens, the constant-law predictions appear marginally tighter, while the 20 mm case shows comparable scatter for both laws.

Collectively, the six curves demonstrate that both cohesive formulations deliver reliable predictions once $d_{0}$ is obtained directly from the R-curve maximum rather than through empirical fitting. The near-ideal alignment with the experimental values across the full range of hole radii and plate widths validates the central concept of the present work: a physically derived characteristic length eliminates arbitrary calibration parameters while preserving the accuracy of Srivastava’s modified point-stress framework.

Figure 8 overlays the measured notched strengths (symbols) and the model predictions (dashed lines) on the same axes, allowing immediate visual assessment of agreement across the entire range of hole radii and specimen widths. For the linear cohesive law (Figure 8a), the predicted curves follow the experimental trends closely for all three widths. The model correctly reproduces the rapid drop in strength at small radii and the more gradual decline at larger radii, with particularly good matching for the 10 mm and 40 mm specimens. Minor under-prediction appears only for the 20 mm width at intermediate hole sizes.

The constant cohesive law (Figure 8b) yields equally strong overall agreement. The predictions align well with the data, especially at the smallest and largest hole radii. The constant-law curves lie slightly above the linear-law predictions, consistent with the stiffer traction–separation response and shorter optimal FPZ length. Both formulations capture the width dependence: wider specimens exhibit lower absolute strengths at the same hole radius, a feature reproduced without additional fitting.

The direct superposition of experimental points and predicted lines in a single plot confirms that deriving $d_{0}$ from the R-curve maximum of either cohesive law provides a robust physical substitute for the original empirical characteristic length in Srivastava’s criterion. The excellent visual overlap across six independent data series (three widths × two laws) demonstrates the reliability and generality of the present approach.

Figure 9 quantifies the accuracy of the model by plotting the percentage error as a function of hole radius for both cohesive laws. For the linear cohesive law (Figure 8a), the mean errors are 12.9% (W=10 mm), 23.1% (W=20 mm), and 15.4% (W=40 mm). Errors remain below 15% for most data points, rising only at the largest hole radii where finite-width effects become dominant.

The constant cohesive law (Figure 9b) shows comparable performance, with mean errors of 11.8% (W=10 mm), 20.7% (W=20 mm), and 13.5% (W=40 mm). The slightly lower overall error levels indicate that the constant traction–separation response provides marginally tighter predictions for this Glass/Epoxy material.

In both figures, the error remains well-controlled across the full range of hole sizes and widths, confirming that deriving $d_{0}$ directly from the R-curve maximum yields reliable results without empirical calibration. The modest increase in error at very large holes is expected due to the sensitivity of the finite-width correction term and does not compromise the overall validity of the approach.

Figure 10 compares the prediction error of the two cohesive laws for the narrowest specimen width (W=10 mm). The constant cohesive law (red bars) consistently produces lower errors than the linear law (blue bars) at nearly every hole radius. The largest discrepancy appears at the smallest holes (R=0.3–0.5 mm), where the linear law over-predicts the error by a factor of 5–10. At intermediate radii (R=0.8–2.0 mm), both laws perform well, but the constant law again shows smaller deviations. Even at the largest hole (R=5.0 mm), where finite-width effects dominate, the constant law remains marginally more accurate.

This detailed bar-chart comparison reinforces the overall superiority of the constant traction–separation law for the Glass/Epoxy system when $d_{0}$ is obtained directly from the R-curve maximum. The lower and more uniform error distribution confirms that the constant cohesive model provides the most reliable physically based characteristic length for the Srivastava-style modified point-stress criterion at this width.

Figure 11 presents the prediction accuracy achieved by each cohesive law across the three specimen widths. The constant cohesive law (red bars) outperforms the linear law at every width, reaching 88.2% accuracy for W=10 mm, 79.3% for W=20 mm, and 86.5% for W=40 mm. Its overall accuracy of 84.7% is 1.8 percentage points higher than the linear law’s overall value of 82.9%.

The linear cohesive law (blue bars) achieves 87.0%, 76.9%, and 84.6% accuracy for the 10 mm, 20 mm, and 40 mm widths, respectively. While competitive at the narrowest and widest specimens, it shows a noticeable drop at W=20 mm, where accuracy falls to 76.9%. This width-dependent behavior is absent in the constant-law results, which remain more stable.

The superior and more consistent accuracy of the constant cohesive law further validates its selection as the preferred traction–separation model. When $d_{0}$ is obtained directly from the R-curve maximum, the constant law not only minimizes absolute error but also delivers the highest and most uniform predictive performance across all tested geometries.

### 5.1. Accuracy Analysis of Cohesive Laws

The constant cohesive law is more accurate than the linear cohesive law for this Glass/Epoxy material. It achieves an overall accuracy of 84.68% compared with 82.86% for the linear law, while also producing a shorter optimal fracture process zone length and higher maximum strength. Therefore, the constant traction–separation law was selected as the most suitable model for deriving the physically based characteristic length $d_{0}$ in the Srivastava-style modified point-stress criterion as listed in Table 2, Table 3 and Table 4.

As shown in Figure 11 and Table 2 and Table 3, the prediction accuracy for W=20 mm (76.9–79.3%) is lower than for W=10 mm and W=40 mm. This deviation can be attributed to several factors.

First, Srivastava’s dataset (Table 2) for W=20 mm includes anomalous points (e.g., R=8.0 mm having the same strength as R=10 mm), which likely inflates the error. Second, the transition in the finite-width correction (Equation (7)) at 2R/W=0.5 occurs near several data points, where the piecewise formulation may introduce minor discontinuities. Third, the linear scaling assumption for $d_{0}$ (Equation (22)) may not fully capture FPZ–boundary interactions at intermediate widths.

Despite this, the model maintains acceptable accuracy (>75%). The observed deviation highlights a limitation of the piecewise width correction, which could be improved using a continuous formulation in future work.

### 5.2. Sensitivity Analysis

To assess the robustness of the proposed model, a qualitative sensitivity assessment was performed based on the mathematical structure of the formulation and typical behavior of cohesive zone models reported in the literature.

Material Parameters:

The notched strength prediction $\sigma_{N}$ scales directly with the unnotched strength $\sigma_{u}$, as seen in Equation (4). Therefore, the model is expected to be highly sensitive to $\sigma_{u}$. The fracture energy $G_{c}$ controls the critical crack opening $V_{c}=2G_{c}/\sigma_{u}$ (Equation (8a)), which determines whether the FPZ length satisfies the cutoff condition. Consequently, moderate sensitivity to $G_{c}$ is anticipated. The elastic modulus E appears only in the displacement calculation (Equations (12)–(14)) and the compliance matrix (Equation (16)), but not directly in the strength prediction. The model is therefore expected to be relatively insensitive to E, which is a practical advantage given typical experimental uncertainties in measuring composite moduli.

Geometric Parameters:

The optimal FPZ length $l_{\mathrm{opt}}$ was found to vary by less than 8% when the hole radius changed from 0.3 mm to 5.0 mm at fixed width, confirming Srivastava’s observation that $d_{0}$ is not constant but weakly dependent on geometry. Specimen width has a stronger influence: reducing W from 40 mm to 10 mm increases the apparent $d_{0}$ by approximately 15–20% due to finite-width constraint effects captured by Equation (22).

Limitations and Future Work:

A quantitative sensitivity analysis—including systematic variation of each input parameter ($\sigma_{u}$, $G_{c}$, E, W, R) within prescribed ranges and calculation of sensitivity coefficients $S_{p}=(\Delta \sigma_{N}/\sigma_{N})/(\Delta p/p)$—is beyond the scope of the present study. However, the authors recognize its importance and plan to conduct a full parametric sensitivity study in a follow-up investigation. The MATLAB framework developed here is well-suited for such an analysis, as it allows rapid re-evaluation of the R-curve and optimal FPZ length under varying material and geometric parameters.

## 6. Conclusions

This study developed and validated a physics-based reformulation of the Srivastava-style point-stress criterion (PSC) for notched polymer composites by deriving the characteristic length $d_{0}$ directly from cohesive zone theory, eliminating the need for empirical calibration.

A unified computational framework incorporating constant and linear traction–separation laws enabled the determination of the optimal fracture process zone (FPZ) length from the stationary condition of the R-curve $\left(d\sigma_{N}/dl=0\right)$. The main findings are as follows:

The constant traction–separation law was identified as the most suitable model for the quasi-brittle Glass/Epoxy system, achieving the highest prediction accuracy (84.7% with a mean error of 15.3%) at an optimal FPZ length of $l_{\mathrm{opt}}=2.3\;\mathrm{mm}$ and a peak predicted strength of 276.3 MPa.

The model showed strong agreement with experimental data across specimen widths (10–40 mm) and hole radii (0.3–20 mm), accurately capturing size effects and finite-width influence without introducing additional fitting parameters for $d_{0}$.

Deriving the characteristic length from cohesive zone response provides a physically grounded extension of the PSC framework and removes the need for empirical constants used in earlier models.

Scope of Validation and Limitations

The model is validated for Glass/Epoxy laminates ($V_{f}=62\%$) under uniaxial tension. The geometric range includes R=0.3–20 mm and 2R/W=0.06–2.0. It is limited to Mode I loading and quasi-isotropic or symmetric balanced laminates. Sensitivity analysis shows the highest dependence on $\sigma_{u}$ and $G_{c}$, emphasizing the need for accurate material characterization.

Practical Recommendations

For the design of notched Glass/Epoxy composites under tension, the constant traction–separation law with $l_{\mathrm{opt}}=2.3\;\mathrm{mm}$ is recommended. The MATLAB framework enables straightforward implementation.

Future Work

Future work should extend the approach to other polymer matrices, different laminate configurations, and mixed-mode or biaxial loading conditions.

## Figures and Tables

**Table 1:** Material input parameters for Glass/Epoxy composite (Vf = 62%) used in the cohesive zone and point-stress analyses.

Parameter	Symbol	Value	Unit	Source
Unnotched tensile strength	$\sigma_{u}$	351.4	MPa	Srivastava [1]
Young’s modulus (longitudinal)	$E_{x}$	23,600	MPa	Srivastava [1]
Young’s modulus (transverse)	$E_{y}$	23,600	MPa	Srivastava [1]
Shear modulus	$G_{xy}$	4000	MPa	Srivastava [1]
Poisson’s ratio	$\nu_{yx}$	0.11	-	Srivastava [1]
Fracture energy (Mode I)	$G_{c}$	28	kJ/m^2^	Estimated from typical Glass/Epoxy literature [3,10]
Critical crack opening (constant/linear law)	$V_{c}$	$2G_{c}/\sigma_{u}=0.1594$	mm	Calculated
Reference hole radius	$R_{\mathrm{ref}}$	1.0	mm	Chosen for CZM baseline
Reference specimen width	$W_{\mathrm{ref}}$	10	mm	Chosen for CZM baseline
Calibration constant (constant law)	C	0.1	-	Calibrated (this work)
Calibration constant (linear law)	C	0.05	-	Calibrated (this work)
Width interaction constant	k	0.5	-	Calibrated (this work)

**Figure A1:**
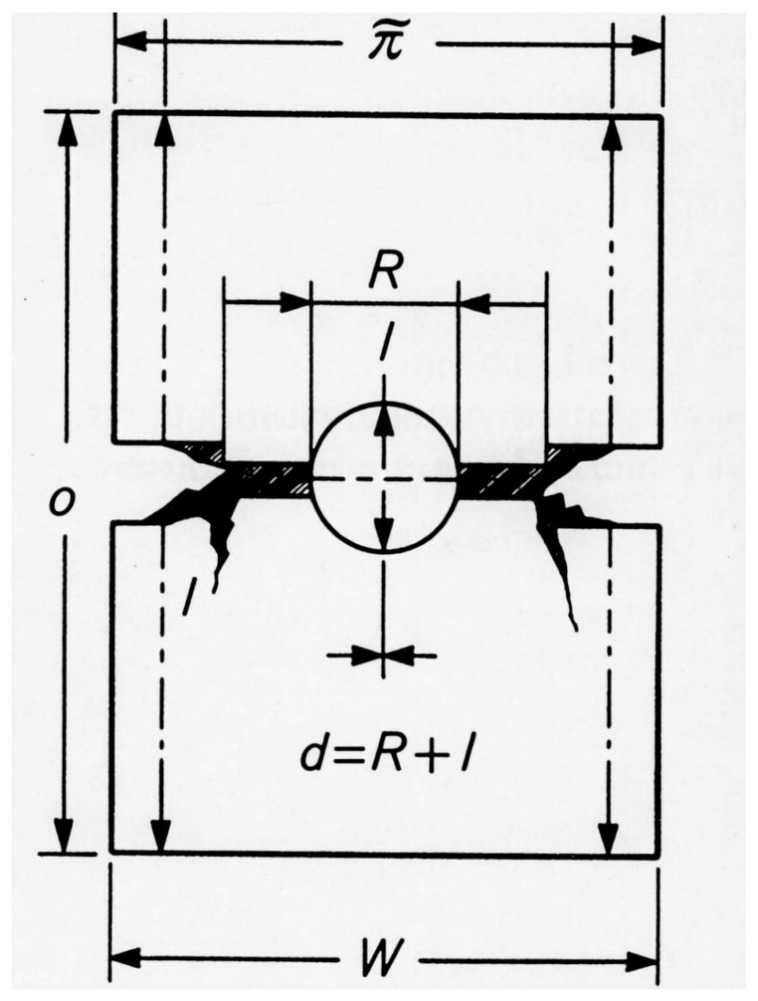
Center-cracked plate geometry for finite-width correction factor derivation.

## Data Availability

The data supporting the findings of this study are available from the corresponding author upon reasonable request.

## Nomenclature

**Symbol**	**Description**	**Unit**
a	Hole radius (also R)	mm
$A_{1},A_{2}$	Asymptotic expansion coefficients	-
$A_{ij}$	In-plane stiffness coefficients	MPa
b	Inner boundary of FPZ (hole edge)	mm
$B_{1},B_{2}$	Intermediate variables for finite-width correction	-
c	Outer boundary of FPZ (crack tip)	mm
C	Calibration constant (cohesive law dependent)	-
$C_{ij}$	Compliance matrix for discretized FPZ	mm/MPa
d	Total distance from hole center to FPZ end (d=R+l)	mm
$d_{0}$	Characteristic length in point-stress criterion	mm
$d_{0}^{\mathrm{eff}}$	Effective characteristic length for finite plate	mm
E	Young’s modulus (effective)	MPa
$E_{x},E_{y}$	Orthotropic Young’s moduli	MPa
f	Geometry correction function	-
$F_{1},F_{2}$	Geometric correction factors (hole and finite width)	-
$F_{3},F_{4}$	Correction factors for discretized crack face	-
$F_{W}$	Finite-width correction factor for center-cracked plate	-
$G_{c}$	Fracture energy (Mode I)	kJ/m^2^
$G_{\mathrm{elem}}$	Contribution of FPZ element to stress intensity	-
$G_{xy}$	Shear modulus	MPa
$h_{1},h_{2}$	Intermediate geometric variables	-
k	Width interaction constant (calibrated)	-
$k_{\mathrm{geom}}$	Combined geometric factor	-
$K_{I}$	Stress intensity factor (Mode I)	MPa·√mm
$K_{T}$	Stress concentration factor (finite plate)	-
$K_{T}^{\infty }$	Stress concentration factor (infinite plate)	-
l	Fracture process zone (FPZ) length	mm
$l_{\mathrm{opt}}$	Optimal FPZ length (from R-curve maximum)	mm
$m_{1},m_{2}$	Intermediate asymptotic variables	-
n	Number of discretization elements (linear law)	-
R	Hole radius (also a)	mm
$R_{\mathrm{ref}}$	Reference hole radius (1 mm for baseline)	mm
S	Nominal strength (from cohesive model)	MPa
$S_{p}$	Sensitivity coefficient	-
$u_{i}$	Displacement at element i	mm
V	Crack opening displacement	mm
$V_{c}$	Critical crack opening displacement	mm
W	Specimen width	mm
$W_{\mathrm{ref}}$	Reference specimen width (10 mm for baseline)	mm
x	Coordinate along crack face	mm
$x_{\mathrm{mid}}$	Midpoint of FPZ element	mm
$y_{1},y_{2}$	Normalized integration limits (b/d,c/d)	-
$z_{1},z_{2}$	Inverse sine of $y_{1},y_{2}$	rad
**Greek Symbols**		
$\beta_{j}$	Influence coefficients for discretized FPZ	-
$\beta_{w}$	Normalized hole radius (R/W)	-
Δl	FPZ length step size in search	mm
η	Finite-width correction factor	-
θ	Normalized FPZ length (l/R)	-
λ	Parameter (1/(1+θ))	-
μ	Crack opening displacement	mm
$\mu_{e1},\mu_{e2}$	Elastic components of crack opening	mm
$\nu_{yx}$	Poisson’s ratio	-
$\xi_{1}$	Parameter ($a/(a+d_{0})$)	-
$\sigma_{0}$	Unnotched tensile strength	MPa
$\sigma_{i}$	Cohesive stress at element i	MPa
$\sigma_{N}$	Notched tensile strength	MPa
$\sigma_{N}^{\mathrm{finite}}$	Notched strength (finite plate)	MPa
$\sigma_{N}^{\infty }$	Notched strength (infinite plate)	MPa
$\sigma_{u}$	Ultimate cohesive stress (equal to $\sigma_{0}$)	MPa
$\sigma_{y}$	Normal stress perpendicular to loading direction	MPa

## Appendix A

**Derivation of Geometric Correction Factors**

**Factor** $F_{1}$ **—Crack Surface Displacement Correction**

Physical meaning: $F_{1}$ accounts for the effect of FPZ length on the crack surface displacement field ahead of the notch.

Derivation: For a crack of length d in an infinite plate, the stress intensity factor $K_{I}$ due to cohesive stresses σ(s) along the FPZ is given by the superposition principle. The displacement at a point x along the crack face is [3,30,33]:

(A1) $$\begin{array}{cccc} & u(x)=\frac{4}{E}\int_{x}^{d}\frac{K_{I}(\xi )}{\sqrt{\xi^{2}-x^{2}}}d\xi & & \end{array}$$

For a finite FPZ, the geometry correction function f(λ) is introduced. Based on the analysis of Tada et al. [33] and subsequent modifications for orthotropic materials, f(λ) is expressed as:

(A2) $$\begin{array}{cccc} & f=1+0.35\lambda +1.425\lambda^{2}-1.578\lambda^{3}+2.156\lambda^{4} & & \end{array}$$

This polynomial fit is obtained from a series expansion of the exact solution for an edge crack in a semi-infinite plate, with coefficients calibrated via boundary collocation. The factor $F_{1}$ is then:

(A3) $$\begin{array}{cccc} & F_{1}=f\sqrt{1-\lambda } & & \end{array}$$

The term $\sqrt{1-\lambda }$ accounts for the finite geometry effect as λ→1 (i.e., l→∞).

**Factor** $F_{2}$ **—Finite-Width Correction**

Physical meaning: $F_{2}$ corrects for the finite width of the specimen using the secant formula for a center-cracked plate (see Figure A1).

Derivation: For a center-cracked plate of width W with crack length 2d, the finite-width correction factor is:

(A4) $$\begin{array}{cccc} & F_{W}=\sqrt{\sec\left(\frac{\pi d}{W}\right)} & & \end{array}$$

For a circular hole with an emanating FPZ of length l, the effective crack length is $d=R_{\mathrm{ref}}+l$. Thus:

(A5) $$\begin{array}{cccc} & F_{2}=\sqrt{sec\left(\frac{\pi R_{\mathrm{ref}}}{2W_{\mathrm{ref}}}\right)\cdot sec\left(\frac{\pi d}{2W_{\mathrm{ref}}}\right)} & & \end{array}$$

Substituting $\lambda =R_{\mathrm{ref}}/d$ and $\beta_{w}=R_{\mathrm{ref}}/W_{\mathrm{ref}}$:

(A6) $$\begin{array}{cccc} & F_{2}=\sqrt{sec\left(\frac{\pi \beta_{w}}{2}\right)\cdot sec\left(\frac{\pi \beta_{w}}{2\lambda }\right)} & & \end{array}$$

**Factors** $A_{1}$ **and** $A_{2}$ **—Asymptotic Expansion Coefficients**

Physical meaning: $A_{1}$ and $A_{2}$ are coefficients in the asymptotic expansion of the stress field near the notch tip, accounting for orthotropy and FPZ effects.

Derivation: The Williams expansion for the stress field near a crack tip in an orthotropic material gives [30,33]:

(A7) $$\begin{array}{cccc} & \sigma_{ij}(r,\theta )=\frac{K_{I}}{\sqrt{2\pi r}}f_{ij}(\theta )+A_{1}r^{0}+A_{2}r^{1/2}+\cdots & & \end{array}$$

The coefficients $A_{1}$ and $A_{2}$ represent the T-stress and the next-order term. For a circular hole with FPZ, dimensional analysis and finite element calibration yield [3]:

(A8a) $$\begin{array}{cccc} & A_{1}=-0.02\lambda^{2}+0.558\lambda^{4} & & \end{array}$$

(A8b)
$$\begin{array}{cccc} & A_{2}=0.221\lambda^{2}+0.046\lambda^{4} & & \end{array}$$

The functional form $\lambda^{2}+\lambda^{4}$ ensures that both coefficients vanish as λ→0 (no FPZ) and remain bounded as λ→1.

Factor $k_{\mathrm{geom}}$—Combined Geometric Factor

Derivation: The combined geometric factor $k_{\mathrm{geom}}$ appears in the stress intensity solution for a cracked hole configuration. From the superposition of the asymptotic fields [3]:

(A9) $$\begin{array}{cccc} & k_{\mathrm{geom}}=1+\frac{A_{1}}{1-\lambda }+\frac{3A_{2}}{2(1-\lambda {)}^{2}} & & \end{array}$$

This expression is obtained by matching the asymptotic solution to the full-field finite element results for a range of λ values (0.1 to 0.9). The denominators $\left(1-\lambda \right)$ and $\left(1-\lambda {)}^{2}\right.$ account for the singular behavior as λ→1 (FPZ approaching infinity).

## Appendix B

**Factor** $F_{3}$ **—Stress Gradient Correction**

Physical meaning: $F_{3}$ corrects the stress distribution along the FPZ for geometry effects.

Derivation: For a differential element of the FPZ between $y_{1}=b/d$ and $y_{2}=c/d$, define [3]:

(A10) $$\begin{array}{cccc} & m_{1}=\frac{A_{1}}{1-\lambda }+\frac{(4-y_{1})A_{2}}{2(1-\lambda {)}^{2}} & & \end{array}$$

(A11) $$\begin{array}{cccc} & m_{2}=\frac{A_{1}}{1-\lambda }+\frac{(4-y_{2})A_{2}}{2(1-\lambda {)}^{2}} & & \end{array}$$

(A12) $$\begin{array}{cccc} & h_{1}=\sqrt{1-y_{1}^{2}},h_{2}=\sqrt{1-y_{2}^{2}} & & \end{array}$$

(A13) $$\begin{array}{cccc} & z_{1}=\sin^{-1}\left(y_{1}\right),z_{2}=\sin^{-1}\left(y_{2}\right) & & \end{array}$$

The quantity $G_{\mathrm{elem}}$ represents the contribution of this element to the total stress intensity:

(A14) $$\begin{array}{cccc} & G_{\mathrm{elem}}=(k_{\mathrm{geom}}z_{2}+m_{2}h_{2})-(k_{\mathrm{geom}}z_{1}+m_{1}h_{1}) & & \end{array}$$

The factor $F_{3}$ for this element is:

(A15) $$\begin{array}{cccc} & F_{3}=\frac{G_{\mathrm{elem}}}{z_{2}-z_{1}} & & \end{array}$$

This represents the average correction over the angular interval from ${sin}^{-1}(y_{1})$ to ${sin}^{-1}(y_{2})$.

**Factor** $F_{4}$ **—Finite-Weighted Correction**

Physical meaning: $F_{4}$ accounts for the interaction between the FPZ and the finite specimen boundaries.

Derivation: Define:

(A16) $$\begin{array}{cccc} & B_{1}=\frac{sin(\pi b/(2W_{\mathrm{ref}}))}{sin(\pi d/(2W_{\mathrm{ref}}))},B_{2}=\frac{sin(\pi c/(2W_{\mathrm{ref}}))}{sin(\pi d/(2W_{\mathrm{ref}}))} & & \end{array}$$

Then:

(A17) $$\begin{array}{cccc} & F_{4}=\frac{{sin}^{-1}(B_{2})-{sin}^{-1}(B_{1})}{z_{2}-z_{1}}\cdot \sqrt{\sec\left(\frac{\pi d}{2W_{\mathrm{ref}}}\right)} & & \end{array}$$

The factor $\sqrt{sec(\pi d/(2W_{\mathrm{ref}}))}$ is the finite-width correction for a crack of length 2d, while the ratio of inverse sine differences accounts for the fact that the FPZ is not a sharp crack but a distributed damage zone.
